# Non-genetic adaptive dynamics for cellular robustness

**DOI:** 10.3389/fgene.2013.00287

**Published:** 2013-12-16

**Authors:** Kumar Selvarajoo

**Affiliations:** ^1^Institute for Advanced Biosciences, Keio UniversityTsuruoka, Japan; ^2^Systems Biology Program, Graduate School of Media and Governance, Keio UniversityFujisawa, Japan

**Keywords:** cell fate, heterogeneity, epigenetics, attractors, robust and adaptive systems

Heterogeneity in molecular expressions is non-longer seen as a nuisance. Recent works have shown that it is required for cellular diversity, in terms of variable responses and for cell fate decisions (Selvarajoo, 2012, 2013). This commentary discusses on the importance of non-genetic heterogeneity for evolutionary robustness.

Darwinian Theory of natural selection, involving heritable traits and mutations, has been long appreciated. However, there are other concepts that have received little attention or a lack of trust. One such theory is Lamarckism, which suggests that acquired characteristics of living systems can be passed over to its offspring without genetic modification. In other words, information regarding the part of a living organism that is often used and unused will help shape the offspring. This hypothesis, originally posed by Jean-Baptiste Lamarck in the early 19th century, has been long debated.

With technological advances in biological experimentations, Lamarckism is gaining interest in the 21st century. This, in part, is due to the appreciation of epigenetics or non-genetic variability observed in living cells. We know epigenetics allow cells to flexibly adapt to changing environments within a lifetime. Lamarckism, on the other hand, suggests the passing on of such flexibility in a “programmed” manner to the offspring. This phenomenon has been tested on different cell types in recent years.

**Figure 1:**
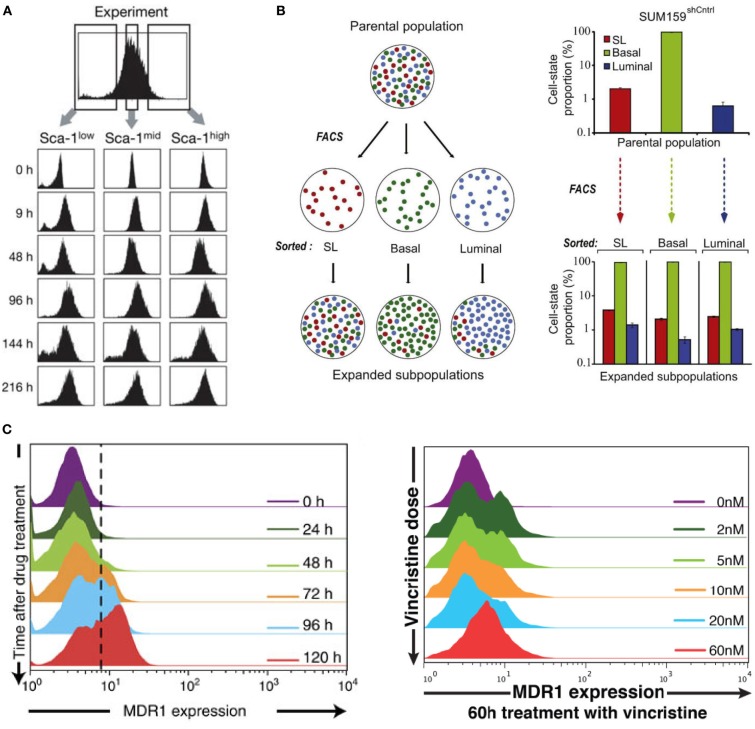
**Non-genetic heterogeneity in living cells.** Re-culturing cells sorted into **(A)** low, medium and high expressions of Sca-1 in mouse hematopoietic cells, **(B)** SL, Basal, and Luminal expressed breast carcinoma cells, returns to original parental metastable or equilibrium state. **(C)** The dynamic (left panel) and VINC dose-dependent (right panel) heterogeneity of HL60 leukemia cells. **(A–C)** are adapted from Chang et al. (2008), Gupta et al. (2011), and Pisco et al. (2013), respectively.

Originally, Sui Huang and colleagues (Chang et al., 2008) studied cell-to-cell variability in mouse hematopoietic progenitor cells, and found the presence of metastable gene expression (attractor) states. Firstly, they showed the expression of Sca-1 protein between cells was heterogeneous and followed a Gaussian-like distribution. Subsequently, they sorted cells using flow cytometry into low, medium and high Sca-1 concentration and re-cultured the cells. Remarkably, the offspring regained the original parental distribution of Sca-1 after 48 h (Figure 1A, Chang et al., 2008). This result suggested the existence of “pre-programmed” states in cells that are regained despite perturbing the cells from their physiological state, independent of selection.

When the sorted cells were perturbed with distinct stimuli, cells with low Sca-1 were more prone for erythroid lineage whereas cells with high Sca-1 were myeloid prone. Thus, non-genetic molecular heterogeneity has a role for diversity in cell differentiation process.

In another work investigating heterogeneity in cancer cells, similar metastable attractor phenomenon was observed (Gupta et al., 2011). Parental population of heterogeneous breast cancer lines were sorted into three subpopulations of mammary epithelial cell states characterized by distinct cell-surface markers: stem-like, basal, and luminal (Figure 1B, Gupta et al., 2011). Culturing the sorted “homogenous” cells over six days gradually returned the offspring to their parental heterogeneous populations. Again, this result demonstrated the presence of strong non-genetic equilibrium state.

More recently, Sui Huang and colleagues investigated the non-genetic ability or plasticity of cancer cells to therapeutic intervention (Pisco et al., 2013). They investigated the cause for drug resistance in HL60 leukemic cells.

Cancer cells can become drug resistant due to the role of certain proteins such as the multidrug resistance protein 1 (MDR1), which is responsible to guide a variety of drugs out of the cells. Untreated leukemic cells showed single distribution of MDR1 expressions with a large majority of cells possessing low expressions and only about 1–2% showing high MDR1 expressions required for drug resistant levels [Figure 1A in Pisco et al. (2013)]. Interestingly, treatment with the drug, vincristine (VINC), showed emergence of bimodal MDR1 distributions, where now a certain number of cells possessed increased MDR1 expressions crucial for drug resistance both with respect to time and with increasing doses of VINC (Figure 1C, Pisco et al., 2013). This emergent behavior is also independent of selection. Re-culturing cells derived from specifically low and high MDR1 expressions both produced offspring that possessed the original parental single modal MDR1 distribution, however, with different relaxation times.

Overall, the experiments from these studies clearly provide evidence that non-genetic heterogeneity is crucial for the adaptability and survivability of living cells to perturbation within their lifetime, and this trait is passed on to their offspring. It is, therefore, conceivable that living systems are not only governed by Darwinian evolutionary principles (longer term), but also through soft inheritance described by Lamarckism (shorter term) for managing survival and adaptation over different time scales.
